# B Cell Lymphoma, Unclassifiable, Transformed from Follicular Lymphoma: A Rare Presentation with Review of the Literature

**DOI:** 10.1155/2015/651764

**Published:** 2015-08-26

**Authors:** Anila Kanna, Swati Agrawal, Kumar Jayant, Varun Kumar Pala, Mohammad Altujjar, Tarik Hadid, Muhammad Khurram

**Affiliations:** ^1^Guntur Medical College, Andhra Pradesh 522004, India; ^2^Nuffield Department of Obstetrics and Gynaecology, University of Oxford, Level 3, Women's Centre, John Radcliffe Hospital, Oxford OX3 9DU, UK; ^3^Transplantation and Hepatobiliary Surgery, University of Iowa Hospitals and Clinics, Iowa City, IA 52242, USA; ^4^Hematology/Oncology Department, Cleveland Clinic Taussig Cancer Institute, Cleveland, OH 44106, USA; ^5^Wayne State University, Detroit, MI 48202, USA; ^6^St. John Hospital and Medical Center, Detroit, MI 48202, USA

## Abstract

B cell lymphoma, unclassifiable, with features of diffuse large B cell lymphoma and classical Hodgkin's lymphoma (BCLu-DLBCL/CHL) is more commonly known as gray zone lymphoma. These cases more often present with mediastinal disease. In this report, we present a very rare case of BCLu-DLBCL/CHL without mediastinal involvement, transformed from follicular lymphoma (FL) to BCLu-DLBCL/CHL. This patient initially presented with a mass in the right neck; biopsy of the lymph node showed predominantly nodular, follicular pattern. Immunohistochemical (IHC) staining of tumor cells expressed positivity for mature B cell markers CD20, CD19, CD10, CD23, CD45, and CD38 but negative for CD5,11c. Hence, diagnosed with FL, he was given rituximab, cyclophosphamide, vincristine, and prednisone (RCVP) regimen, followed by maintenance rituximab. He showed good response. After 2 years, he presented again with a mass in the right side of the neck. Although the needle core biopsy of this mass was suggestive of B cell lymphoma, excisional biopsy showed morphological features of DLBCL as well as foci of histological pattern of CHL. IHC staining expressed positivity for CD20, CD79a, PAX5, and CD15 and CD30 consistent with DLBCL and CHL. He was diagnosed with BCLu-DLBCL/CHL. The patient received “ACVBP” (doxorubicin, cyclophosphamide, vindesine, bleomycin, and prednisone) followed by radiation. BCLu-DLBCL/CHL is clinically an aggressive tumor with poorer outcomes, but our case showed complete response to ACVBP regimen with tumor regression.

## 1. Introduction

B cell lymphoma, unclassifiable, with intermediate features of two different types of lymphomas, diffuse large B cell lymphoma (DLBCL) and classical Hodgkin's lymphoma (CHL), or Burkitt lymphoma (BL) is more commonly called gray zone lymphoma (GZL). According to Revised European-American Classification of Lymphoid Neoplasms (REAL Classification), GZL are characterized as any lymphomas that cannot reproducibly be assigned to a particular diagnosis [1]. The term GZL was first used in 1998 at the Workshop on Hodgkin's Disease and Related Diseases to designate lymphomas at the border of CHL and other entities [2].

It was, in 2008, that World Health Organization (WHO) has introduced GZL for the first time, in the 4th edition of the Classification of Tumors of Hematopoietic and Lymphoid Tissue, as a B cell lymphoma, unclassifiable, with features of both DLBCL and CHL or BL [3]. Since GZL is a new inclusion in the latest WHO classification and also due to its rare occurrence, the diagnosis of this lymphoma is relatively challenging. There is only little reported clinical data regarding its therapy and outcomes as it is a rare entity.

NHL and CHL are morphologically and clinically two distinct neoplasms. Among NHL cases worldwide, mature B cell lymphomas are the most predominant type and these mature B cell lymphomas have various subtypes. Of the several subtypes of these mature B cell lymphomas, diffuse large B cell lymphoma (DLBCL) is the most predominant subtype, accounting for 28% of cases [4]. So far, the frequency of BCLu-DLBCL/CHL cases was observed to be high in western countries and in sub-Saharan Africa and Asian countries; these cases have been less frequently observed [3]. Although BCLu-DLBCL/CHL more commonly present with mediastinal disease, a few cases with nonmediastinal involvement have been reported. CHL and PMBL which are two different entities have better clinical course and prognosis when compared to the combination of these two entities which is BCLu-DLBCL/CHL which has aggressive clinical course and poor outcome [3, 5]. Since the features of BCLu-DLBCL/CHL are intermediate between DLBCL and CHL, the treatment of this lymphoma is challenging. The treatment of DLBCL and CHL has different combination chemoimmunotherapy regimen, from which the appropriate regimen can be chosen according to stage of the disease. The treatment options for DLBCL include R-CHOP (cyclophosphamide, doxorubicin, vincristine, and prednisone, along with anti-CD20 monoclonal antibody rituximab) and R-EPOCH (rituximab, etoposide, prednisone, vincristine, cyclophosphamide, and doxorubicin) [6]. The treatment options for CHL include ABVD (doxorubicin, bleomycin, vinblastine, and dacarbazine), which is the most commonly used regimen, Stanford V (doxorubicin, vinblastine, mechlorethamine, etoposide, vincristine, bleomycin, and prednisone), and BEACOPP (bleomycin, etoposide, doxorubicin, cyclophosphamide, vincristine, procarbazine, and prednisone) [7]. As BCLu-DLBCL/CHL is new entity included in the WHO classification, no optimal management strategy has been proposed up to date.

Here we report a very rare case of BCLu-DLBCL/CHL with features intermediate between DLBCL and CHL without mediastinal involvement and with tumor transformation from follicular lymphoma to BCLu-DLBCL/CHL and present a review of the literature.

## 2. Case Report

**Figure 1:**
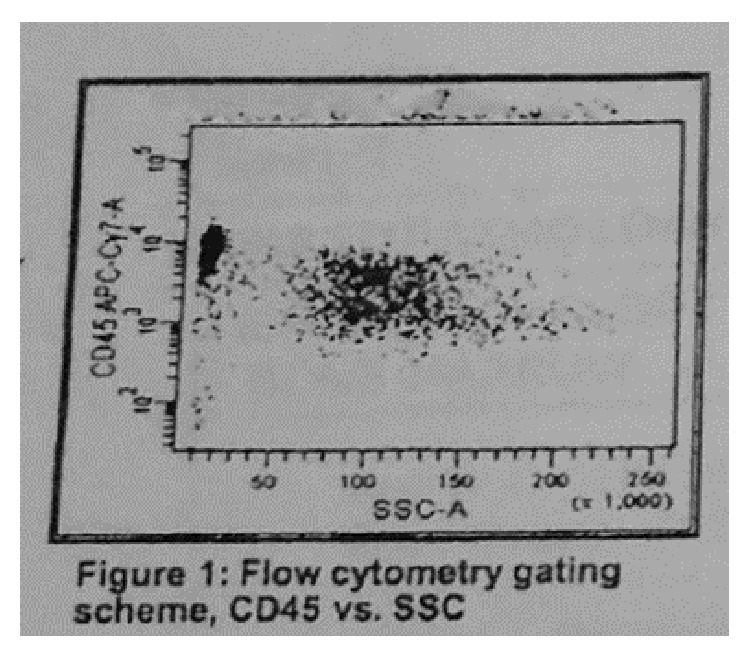
Flow cytometry of the bone marrow biopsy sample, negative.

**Figure 2:**
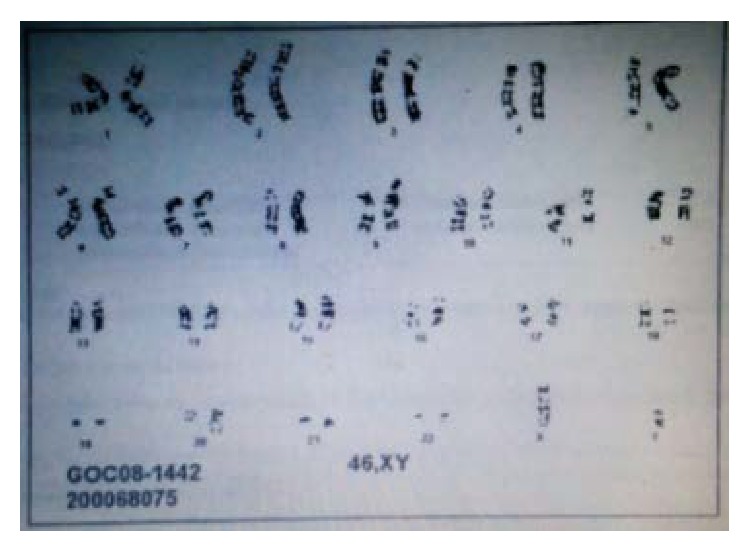
Normal cytogenetic study (bone marrow biopsy sample).

**Figure 3:**
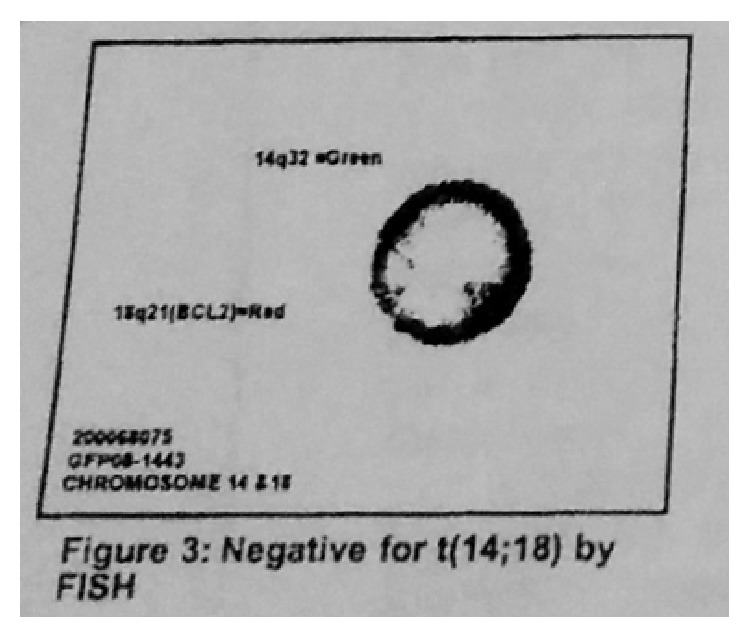
FISH, negative for t(14;18) (bone marrow biopsy sample).

A 56-year-old male presented with mass in the right neck area, asymptomatic at the time of presentation. Biopsy of the lymph node (LN) in the right side of the neck was done. Sections of the lymph node reveal replacement of the normal architecture with a predominantly nodular, follicular pattern. About 10% of the lymph node shows more diffuse proliferation. The follicles showed a mixture of small lymphocytes, larger centroblasts, and scattered follicular dendritic cells, consistent with follicular lymphoma grade 2 of 3. Flow cytometry showed a monoclonal B cell population with kappa cells. Tumor cells, on immunohistochemical staining, expressed positivity for mature B cell markers CD-20, CD-19, CD-10, CD-23, CD-45, and CD-38 but were negative for CD-5,11c. Therefore, he was diagnosed with follicular lymphoma. The bone marrow biopsy was normal cellularity with no evidence of involvement. Flow cytometry results showed no evidence of monoclonal B cell population or an immunophenotypically aberrant T cell population (Figure 1). Cytogenetic study (Figure 2) and FISH analysis for t(14;18) (Figure 3) did not reveal any abnormalities in the bone marrow biopsy sample. Computerised Tomography (CT) scan showed disease both above and below the diaphragm and the largest lymph node was under 3 cms. Hence, he was diagnosed with follicular lymphoma grade 2, stage III. He was placed under observation. In due course, after 1 year, he developed renal insufficiency with an increase in his serum creatinine to 1.7 mg/dL (normal range: 0.6–1.4 mg/dL). CT scan showed enlarged retrocaval LN obstructing left ureter causing left kidney hydronephrosis. Palliative ureteral stenting was done. He was started on chemotherapy after 6 months. He received a total of 6 courses of RCVP (rituximab 375 mg/m^2^ IV on day 1 plus cyclophosphamide 750 mg/m^2^ IV on day 1 plus vincristine 1.4 mg/m^2^ (dose cap at 2 mg) IV on day 1, plus prednisone 40 mg/m^2^ PO on days 1–5; every 21 days for six cycles) and maintenance rituximab was given as per the Hainsworth protocol, that is, four-weekly rituximab every 6 months. Rituximab was given 4 times, as maintenance. After his induction regimen of RCVP, he was followed with Fluorodeoxyglucose positron emission tomography- (FDG/PET-) CT scan every month which showed residual uptake in the right retroperitoneal area on the initial follow-up FDG/PET-CT scan. In the due course, the standardized uptake value (SUV) gradually declined from 5.4 to 0. Also, this patient recovered from the left hydronephrosis and the stent was removed, though underlying chronic renal insufficiency still persisted. Since the completion of rituximab, he has been under surveillance. He responded well to the treatment given and was in remission.

**Figure 4:**
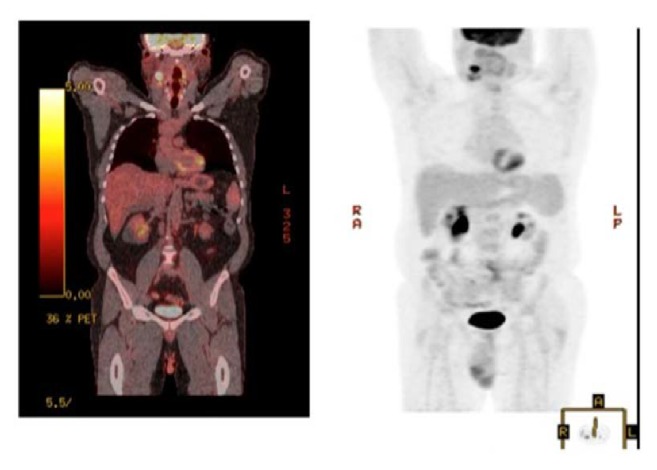
PET scan showing no evidence of any metastasis.

**Figure 5:**
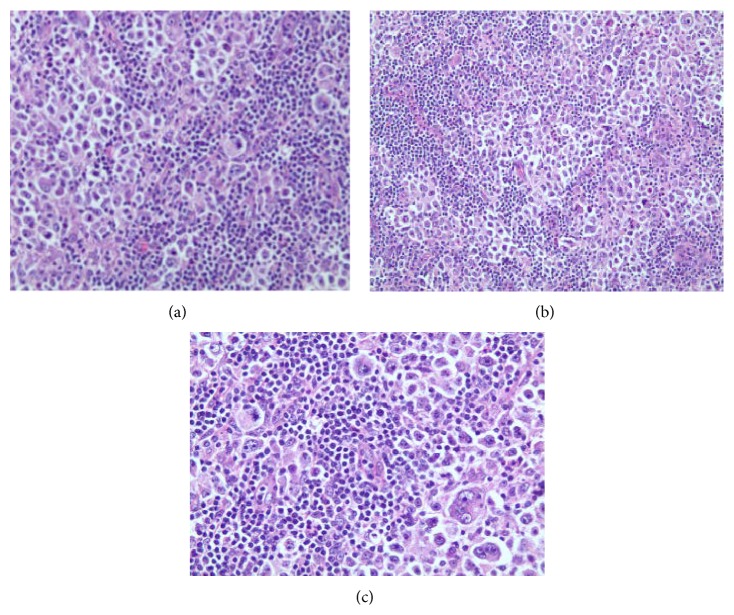
(a, b, c) Hematoxylin & Eosin stain slides showing tumor characteristic.

**Figure 6:**
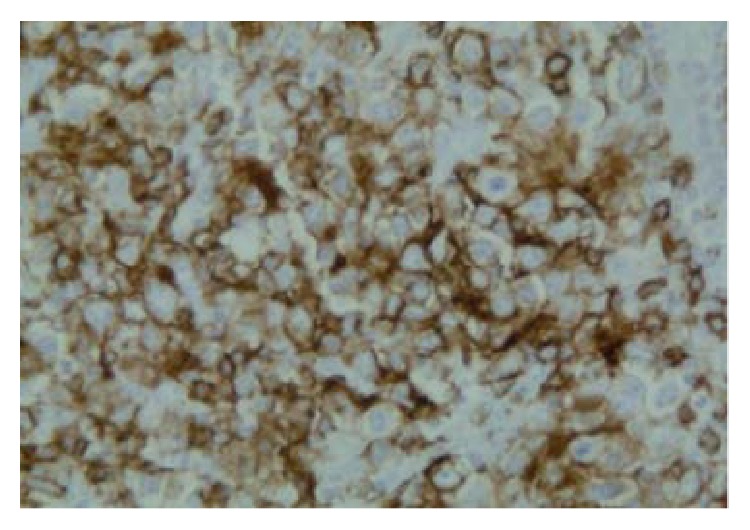
CD 20 positivity suggestive of DLCBL (100x).

**Figure 7:**
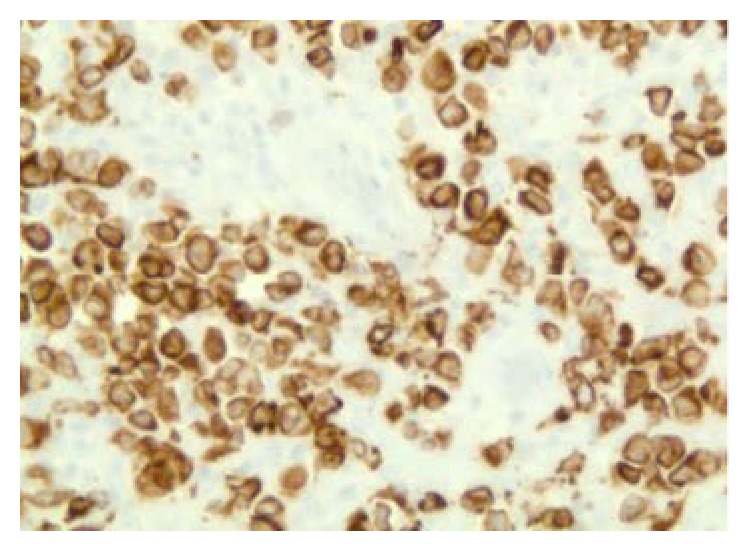
CD 79a positivity suggestive of DLCBL (100x).

**Figure 8:**
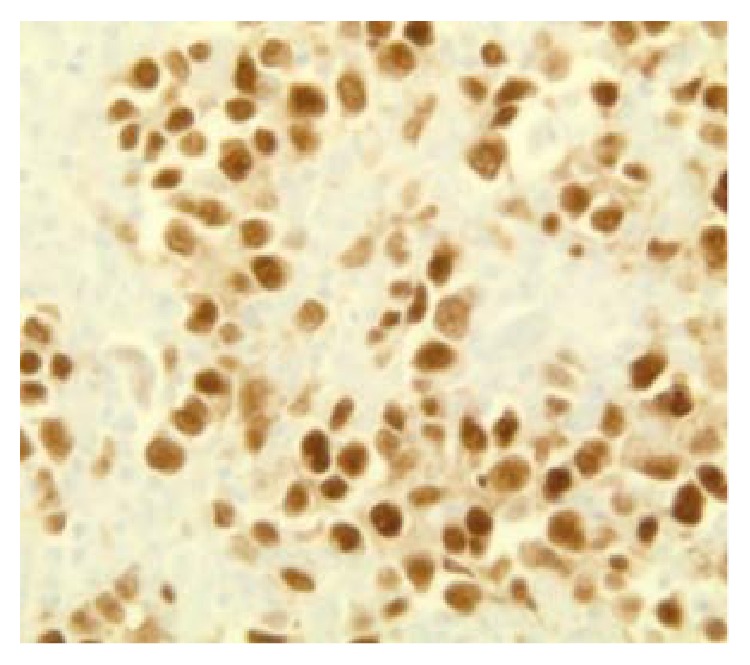
PAX 5 positivity suggestive of DLCBL (100x).

**Figure 9:**
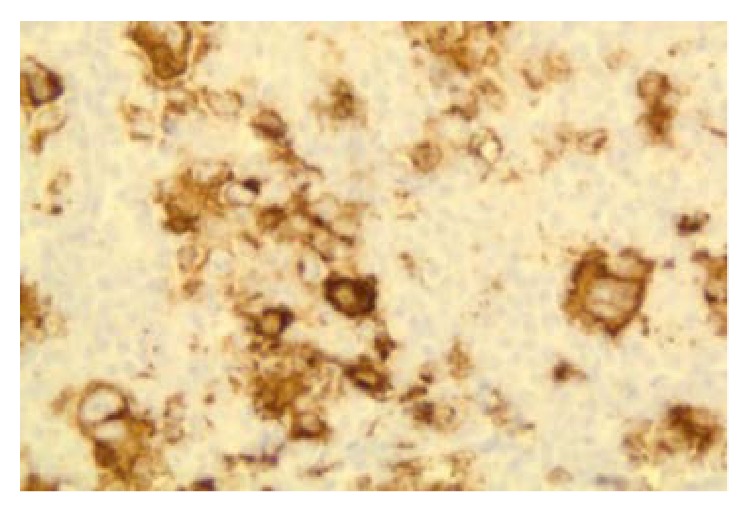
CD15 positivity consistent with CHL (100x).

**Figure 10:**
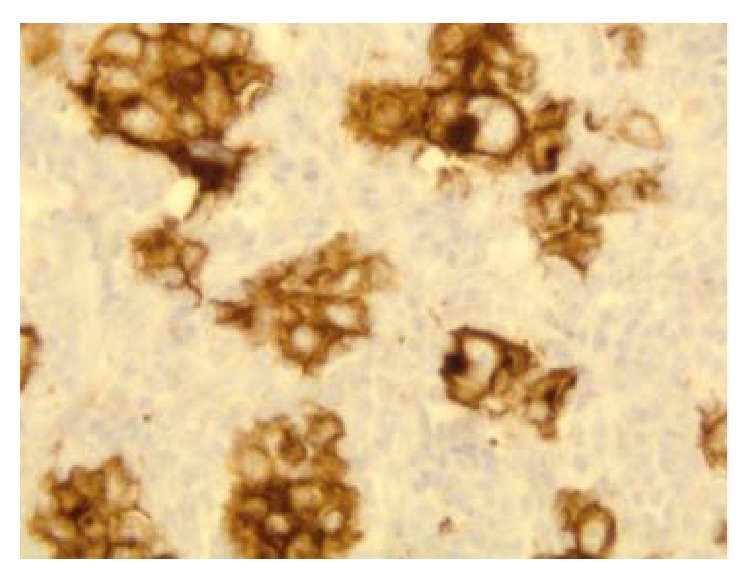
CD30 positivity suggestive of CHL (100x).

**Figure 11:**
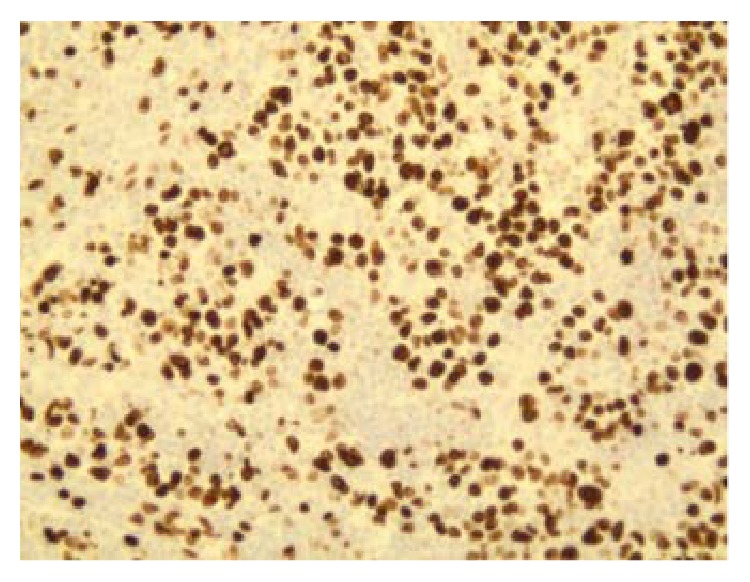
Ki-67 positivity index of malignancy (100x).

After 2 years of follow-up period, he presented again with a mass on the right side of the neck, asymptomatic. Left cervical lymphadenopathy was also present. FDG/PET-CT scan showed multiple hypermetabolic lymph nodes with SUV max uptake of 15.6, measuring 4.4 × 1.9 cm consistent with recurrent lymphoma (Figure 4). Laboratory examination revealed WBC count of 6100/mcL (normal range 3600–11000/mcL), hemoglobin of 15.3 gm/dL (normal range 13.0–16.0 gm/dL), platelets of 1,22,000/mcL (normal range 130000–450000/mcL) and LDH of 155 U/L (normal range 135–225 U/L). Needle core biopsy of the LN on the right side of the neck showed findings suggestive of large B cell lymphoma. However, excisional biopsy showed morphological features of DLBCL as well as foci of histological pattern of CHL. Several large cells were noticed, of which many were binucleated and multinucleated. Prominent mitotic activity and pleomorphism were noted (Figures 5(a), 5(b), and 5(c)). Immunohistochemical staining of the tumor cells showed positivity for CD 20 (Figure 6), CD79a (Figure 7), PAX 5 (Figure 8), bcl-2, and bcl-6+ consistent with DLBCL and CD 15 (Figure 9) and CD 30 (Figure 10) consistent with CHL and negativity for CD3, CD5, CD10, ALK-1 protein, EMA, and Epstein Barr Virus (EBV) (EBER by ISH-EBV- (Epstein-Bar Virus-) Encoded RNA (Ribonucleic Acid) In Situ Hybridization). Ki-67 was extremely high with approximately 80% (Figure 11). CD 45 was equivocal; however, in foci, there was a suggestion of foci of weak positivity. CD 21 highlighted the residual follicular dendritic meshwork of the residual follicles in the lymph node. The histologic pattern shows foci with an appearance of classical Hodgkin's lymphoma, as well as areas with morphological features of a DLBCL. With the histologic features and pattern, as well as the immunohistochemical staining pattern, the findings were more consistent with BCLu-DLBCL/CHL. This tumor showed tumor transformation from follicular lymphoma to BCLu-DLBCL/CHL, which is a very rare condition. Flow cytometry analysis from excisional biopsy showed no evidence of monoclonal B cell population or aberrant T cell antigen expression immunophenotypically. Bone marrow biopsy was negative. Bone marrow cytogenetic analysis noted normal male karyotype 46XY.

In the due course of time, after 9 weeks from the time when the new neck mass was noticed, the neck mass completely resolved compared to the prior measurement, despite no treatment given. FDG/PET-CT which was done to restage the patient showed partial response, with redemonstration of hypermetabolic right cervical adenopathy. The largest node at level 2a measuring 23 × 19 mm in short axis with max SUV 11.1 improved from 15.6 when compared to the prior one. This corresponds to a 5-point node on Deauville scale. Mildly enlarged retrocaval lymph node was noted measuring 1.3 × 1.7 cm, smaller than the prior one, which was not hypermetabolic. Patient was given 3 courses of combination chemotherapy ACVBP regimen given every 3 weeks of doxorubicin (75 mg/m^2^) on day 1, cyclophosphamide (1200 mg/m^2^) intravenously on day 1, vindesine (2 mg/m^2^) on days 1 and 5, bleomycin (10 mg) on days 1 and 5, prednisone (60 mg/m^2^) orally from day 1 to day 5, and intrathecal methotrexate (15 mg) on day 2. Granulocyte-macrophage or granulocyte colony-stimulating factor was administered subcutaneously on days 6 through 13 of each cycle. Rituximab was not given. Bone marrow biopsy after chemotherapy was negative. He was given consolidated field radiation therapy of the right neck. FDG/PET-CT done after treatment showed complete response to ACVBP regimen and consolidated field radiation therapy.

## 3. Discussion

B cell lymphoma, unclassifiable, exhibiting features intermediate of both diffuse large B cell lymphoma (DLBCL) and classical Hodgkin's lymphoma (CHL) is also known as gray zone lymphoma (GZL). Traverse-Glehen et al. was the first group to describe GZL as a type of lymphoma with combining features of CHL and DLBCL, mostly primary mediastinal large B cell lymphoma (PMBL) in the year 2005 [8].

In the present report, we describe a case of BCLu-DLBCL/CHL with intermediate features of DLBCL and CHL without mediastinal involvement, transformed from follicular lymphoma to BCLu-DLBCL/CHL. Although the needle core biopsy was suggestive of B cell lymphoma, the findings of excisional biopsy showed morphological features of DLBCL as well as foci of histological pattern of CHL. Immunohistochemical staining of the tumor cells expressed positivity for CD 20, CD79a, PAX 5, CD 15, and CD 30 consistent with DLBCL and CHL. This patient's findings indicated the intermediate features of both DLBCL and CHL. Therefore, this patient was diagnosed with BCLu-DLBCL/CHL.

Majority of the patients with BCLu-DLBCL/CHL present in the early stage of disease with mediastinal involvement, but a few cases which present with the nonmediastinal disease have been documented. We present a review of literature of all reported cases of BCLu-DLBCL/CHL to better define its presenting clinical characteristics, treatment, and outcome.

BCLu-DLBCL/CHL is more common in young men and is usually associated with mediastinal disease [3]. According to Eberle et al. [9], in a study of 33 cases of gray zone lymphoma, 73% had mediastinal involvement. Median age was 55 and 29.5 years, without and with mediastinal involvement, respectively, in this study. According to Nadeem et al. [10], in a study of 49 cases, 89% showed mediastinal disease, and median age was 33.5 years and mediastinal disease was more predominant in males. According to Evens et al. [11], 96 cases were analysed, of which 55% showed nonmediastinal involvement. In this study, the median age of presentation was 37 and 50, with and without mediastinal disease, respectively. Mediastinal involvement had bulk mass in 42% of the cases whereas only 8% cases had bulk mass in nonmediastinal disease. The findings of these reports were consistent with our case, a 54-year-old male patient presenting without mediastinal disease.

Our report describes a rare clinical presentation of BCLu-DLBCL/CHL; the patient was a 54-year-old male without mediastinal involvement, showing tumor transformation from follicular lymphoma to BCLu-DLBCL/CHL. According to previous report [12], DLBCL transformed from a follicular lymphoma, with shared histopathological features of CHL and DLBCL, was represented. The transformed tumor cells expressed CD30 positivity along with OCT-2 and PAX5 in the RS-like cells. Immunohistochemical staining of the transformed tumor cells of our case expressed positivity for CD 20, CD79a, PAX 5, bcl-2, and bcl-6+, consistent with DLBCL and CD 15 and CD 30 consistent with CHL, similar to the case mentioned above.

Hodgkin Reed Sternberg (HRS) cells show strong expression of BSAP/PAX5 and weak or no expression of OCT-2 or BOB.1. On the contrary, large neoplastic cells with a strong expression of OCT-2 and/or BOB.1 are more typical of B cell lymphoma [13]. According to the study of 10 cases reported by Gualco et al. [14], the immunohistochemical findings were positive in all cases for CD 30, CD 20, CD45, and PAX5, 70% positive for CD79a, 50% positive for OCT-2, and 40% positive for CD15, BOB.1, and bcl-6. In our case, the cells on immunohistochemical staining expressed PAX5, CD20, CD79a, CD 15, and CD 30.

EBV-positive DLBCL more commonly occurs in the elderly and present with extranodal disease and is histopathologically characterized by atypical large B cells, with HRS-like cells in the polymorphous inflammatory background, similar to CHL and BCLu-DLBCL/CHL [3, 15]. Our case was negative for EBER-ISH confirming EBV negativity.

The clinical course of BCLu-DLBCL/CHL is more aggressive with poorer outcomes than either CHL or PMBL [3]. However, there is no standard treatment, and optimal management is yet to be determined. In our literature review, there are only few reports on the efficacy of combined chemotherapy and radiation therapy. In our case, the patient has shown complete response to 3 courses of ACVBP therapy and radiation with tumor regression.

From our extensive review of literature on nonmediastinal BCLu-DLBCL/CHL (Table 1), the median age of presentation is 52 years, predominantly seen in males. Most of the cases were positive for CD 20 and CD 30. A few cases with EBV positivity have been reported. Many different combination chemotherapies have been given like ABVD, BEACOPP, R-BEACOPP, CHOP+/−R, EPOCH+/−R, and ACVBP. Very few cases had complete response to treatment and our case is one among them. In our study, it is also observed that most of the cases showed CD 20 and CD 30 positivity. Therefore, further studies on the role of rituximab and brentuximab vedotin (antibody-drug conjugate targeting CD 30) in the treatment of this type of lymphomas can be considered.

## 4. Conclusion

We reported a case of BCLu-DLBCL/CHL without mediastinal involvement, transformed from follicular lymphoma to BCLu-DLBCL/CHL. BCLu-DLBCL/CHL is clinically an aggressive tumor with poorer outcomes, but our case showed complete response to ACVBP regimen with tumor regression confirmed with Fluorodeoxyglucose positron emission tomography- (FDG/PET-) CT scan. As these lymphomas are rare and only few cases have been reported, further studies are required to better understand the nature of the disease and propose an optimal management strategy for this type of lymphoma.

## Figures and Tables

**Table 1:** A review of reported cases of nonmediastinal BCLu DLBCL/CHL.

Study	Cases	Age/sex	Site	Initial diagnosis	Immunohistochemical staining	Rebiopsy diagnosis	Treatment	Outcome
Index case	1 case	56/M	Right neck mass	Follicular lymphoma	CD20+, CD15+, CD30+, CD79a+, PAX5+, bcl-2, and bcl-6+; Ki-67 80% Negative for CD3, CD5, CD10, ALK-1 protein, EMA, and EBV (EBER by ISH) Cyclin D1 equivocal, CD21+ Large cells binucleated and multinucleated were noted	B cell lymphoma, unclassifiable	ACVBP + radiation	Good response

Iwaki et al. [16]	1 case	78/F	Ileocecal tumor	Nodular sclerosis cHL	+ for CD30, CD20, CD79a, PAX5, BOB.1, and OCT-2, but negative for CD15 Increased level of soluble IL-2 receptor	BCLu-DLBCL/CHL	ABVD	Partial response

Wang et al. [17]	1 case	67/F	Left supraclavicular LN, right axillary LN, bilateral inguinal LAD	NA	CD30 and CD15+; CD20−, PAX5, and CD45 were down-regulated; EBV+	EBV + DLBCL and EBV + CHL	NA	NA

Eberle et al. [9]	1 case	55/M	Neck LN	NA	CD20+, CD30+, CD15, CD79a+, BOB.1+, Oct-2+, p63−, cyclin E−, and HLA-DR4	GZL	NA	NA

Eberle et al. [9]	1 case	67/F	Neck mass	NA	CD20+, CD30+, CD15−, CD79a+, BOB.1+, Oct-2+, p63+, cyclin E+, and HLA-DR4	GZL	NA	NA

Eberle et al. [9]	1 case	58/F	Inguinal LN	NA	CD20+, CD30−, CD15, CD79a+, BOB.1+, Oct-2+, p63−, cyclin E−, and HLA-DR4	GZL	NA	NA

Eberle et al. [9]	1 case	26/M	Neck mass	NA	CD20+, CD30+, CD15+, CD79a+, BOB.1+, Oct-2+, p63−, cyclin E+, and HLA-DR3	GZL	NA	NA

Eberle et al. [9]	1 case	91/F	Axillary LN	NA	CD20−, CD30+, CD15+, CD79a+ BOB.1+, Oct-2+, p63+, cyclin E+, and HLA-DR4	GZL	NA	NA

Eberle et al. [9]	1 case	24/F	Cervical LN	NA	CD20+, CD30+, CD15+, CD79a+, BOB.1+, Oct-2+, p63−, cyclin E+, and HLA-DR4	GZL	NA	NA

Eberle et al. [9]	1 case	48/F	Axillary LN	NA	CD20+, CD30+, CD15+, CD79a+, BOB.1+, Oct-2+, p63+, cyclin E+, and HLA-DR1	GZL	NA	NA

Eberle et al. [9]	1 case	85/M	Axillary mass	NA	CD20+, CD30+, CD15− CD79a+, BOB.1+, OCT2+, p63−, cyclin E+, and HLA-DR4	GZL	NA	NA

Eberle et al. [9]	1 case	25/F	Neck LN	NA	CD20− CD30+, CD15+, CD79a−, BOB.1−, Oct-2−, p63−, cyclin E−, and HLA-DR3	GZL	NA	NA

Quintanilla-Martinez et al. [12]	1 case (under subheading: “Classical Hodgkin's lymphoma with immunophenotypic deviations”)	38 yo	Axillary LN	NA	CD20+, PAX5−, OCT-2−, and CD79a−	CHL morphology but strong CD20 expression in the RS cells	NA	NA

Gualco et al. [14]	10 cases	37 y median age	8 mediastinal, 2 extramediastinal	NA	CD30+ (100%), CD20+ (100%), CD45+ (95%), CD15+ (40%), BOB1+ (40%), OCT2 (50%+, 30%), PAX5+ (90%), bcl-6 (40%), P63 (50%+), and CD79a (70%+)	B cell lymphoma, unclassifiable	combination chemotherapy for non-Hodgkin's lymphomas	Good response

García et al. [5]	9 cases	Young males	3 extramediastinal, 6 mediastinal	NA	CD30+, CD15+ (6/9) CD45RB+, CD20+, CD79a+, OCT2+, and EBV (2/9)	Shared features of DLBCL and classical HL	NA	NA

Nadeem et al. [10]	49 cases; 11% extramediastinal	33.5 Y median age; M > F	11% extramediastinal, 89% mediastinal	NA	CD20+ (90%), CD30+ (95%), 63% CD15+, and EBV+ (9/22 patients)	GZL	Initial cHL regimens (BEACOPP, ABVD) or DLBCL regimens (CHOP, EPOCH, with or without rituximab)	7 pts received ABVD, 57% NR and 43% PR; no CRs 7 pts received CHOP; 5 had clinical outcome data available; 80% CR (received consolidation radiation therapy) and 20% PR 7 pts received autologous SCT and 2 of them had relapse in 6 months

Zarate-Osorno et al. [18]	9 cases: 7 follicular, 1 DLBCL, and 1 large cell immunoblastic	54-year median age	7 (primarily nodal) Name of node not specified in the article	NHL	RS and Hodgkin's cells were LeuM1 or BerH2+ and LCA− in 8/9 biopsies	HD	4 chemotherapies 3 chemotherapies + radiation	NA
